# A meta-analysis of the effects of community-based treatments for asthma in children

**DOI:** 10.1186/s12887-025-06048-y

**Published:** 2025-10-02

**Authors:** Yanyan Li, Ming Ren, Yajuan Wang, Xiaomi Qi, Hui Wang, Na Liang, Tianyang Ren, Lihong Zhang

**Affiliations:** 1 Department of Pediatrics, Baoding First Central Hospital, Hebei, 071000 China; 2 Department of English, China Foreign Affairs University, Beijing, 100091 China

**Keywords:** Asthma-connected emergency department visits, Hospitalizations, Duration of asthma symptoms, Community intervention, Nighttime asthma symptoms

## Abstract

**Background:**

The aim of the study was to appraise and compare the influence of community‑based interventions (C-BIs) for childhood asthma.

**Methods:**

We examined meta-analysis data and employed dichotomous or continuous random or fixed-effect models to obtain odds ratios (ORs) and mean differences (MDs) with 95% confidence intervals (CIs). This review examined 13 papers that included a total of 8824 people with asthma.

**Results:**

Children with C-BI had significantly lower asthma-connected emergency department visits (OR, 0.29; 95% CI, 0.22–0.39, *p* < 0.001), hospitalizations (OR, 0.24; 95% CI, 0.15–0.40, *p* < 0.001), duration of asthma symptoms (MD, -2.56; 95% CI, -2.84- -2.28, *p* < 0.001), nighttime asthma symptoms (MD, -2.14; 95% CI, -2.94- -1.34, *p* < 0.001), and use of bronchodilator (OR, 0.28; 95% CI, 0.16–0.51, *p* < 0.001), and higher use of asthma action plan (OR, 8.87; 95% CI, 3.85–20.45, *p* < 0.001) compared to children without C-BI in asthma.

**Conclusions:**

Children with C-BI had significantly lower asthma-related emergency department visits, hospitalization duration of asthma symptoms, night-time asthma symptoms, and use of bronchodilators, and a higher use of asthma action plans compared to children without C-BI in asthma. Nevertheless, due to the limited number of studies involved in the comparisons, their values warrant careful consideration.

**Supplementary Information:**

The online version contains supplementary material available at 10.1186/s12887-025-06048-y.

## Introduction

339 million people worldwide suffer from asthma, making it a serious public health issue ^1^. Asthma ranks 16th in terms of disability-adjusted life years lost and 28th in terms of illness burden [1]. The 2025 Global Initiative for Asthma (GINA) report highlights several social risks related to asthma, particularly focusing on the impact of climate change and access to affordable, effective treatment. These include increased morbidity due to extreme weather events, potential disruptions in healthcare access, and disparities in treatment availability, especially in low- and middle-income countries [2]. Asthma continues to be a cause of avoidable emergency department visits and hospital admissions in children, notwithstanding advances in disease understanding and accessibility of efficient therapies [3]. Numerous factors contribute to poor success in pediatric asthma control, such as medication noncompliance issues, insufficient asthma education, ineffective environmental trigger mitigation, poor coordination within and between healthcare providers, etc [4]. Asthma-connected health outcomes in children can be improved by creating and implementing an efficient community-based strategy, as causes of these issues frequently lay outside acute care system. Most community-based initiatives created in the past few decades to enhance pediatric asthma tactics have primarily concentrated on a single intervention, such as teaching children and caregivers how to manage their asthma in a specific community context, such as their home or school. Reports on these therapies’ efficacy, however, haven’t always been reliable [5]. Asthma education alone significantly decreased the likelihood of emergency department visits for asthma, but its influence on hospital admissions and vital care physician visits was not statistically significant [5]. This is most likely because asthma is a complicated illness with many underlying causes, including social, environmental, and behavioral determinants of health in addition to physiological ones. To improve health consequences for children with asthma, a combination of interventions that bridge the gap between hospital- and community-based services and address social behavioral, and physiological features of asthma is required. There hasn’t been a thorough analysis of recent data or an attempt to measure the efficacy of these more all-encompassing community-based strategies that link various stakeholders.

## Method

### Design of examination

The meta-analyses were assessed and joined with epidemiological statements utilizing a predefined procedure. Several databases were accessed in gathering and analyzing data. These datasets were applied to collect analyses that compared and gauged the influence of community-based interventions (C-BIs) for childhood asthma [6]. 

## Data pooling

**Fig. 1:**
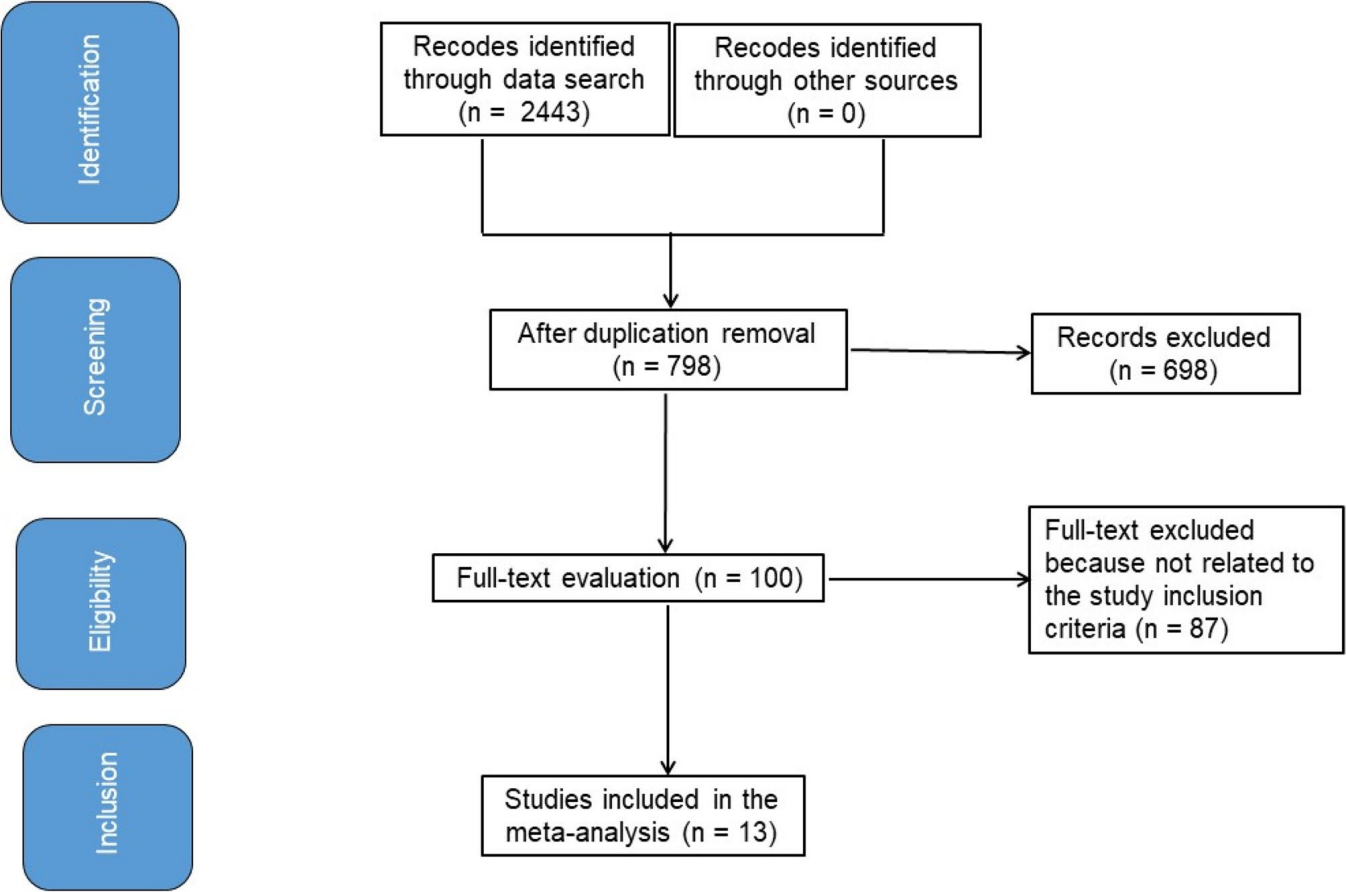
Schematic diagram of examination procedure

It was discovered that C-BI produced several clinical results. This research investigated the primary result of the inclusion parameter. Language problems were not taken into account throughout the inclusion of research or the screening procedure for potential participants. No limitations existed on the number of volunteers available for research. As letters, reviews, and editorials do not provide a position in meta-analysis, we excluded this type from our compilation. Figure 1 illustrates the complete inspection identification process [7]. 

## Eligibility of included studies

The effect of C-BIs for childhood asthma was studied. Only examinations that talked about how interferences influenced the occurrence of different clinical results were encompassed in the sensitivity analysis. Subclass and sensitivity analyses were implemented by associating numerous subtypes with interference groups [8]. 

## Inclusion and exclusion criteria

### Inclusion criteria and exclusion criteria

When the inclusion criteria were satisfied, the literature was combined into the study:

1. The research was a randomized controlled study, observational, retrospective, and prospective.
2. Children with asthma were investigated as elect subjects.
3. The interference incorporated community-based interventions.
4. The study demonstrates the eminent effect of community-based interventions for childhood asthma.

The exclusion of non-comparative study designs occurred.

### Identification of studies

A protocol of search algorithms was established and specified by PICOS principle [9], which states: P (population) Children with asthma; C-BI was “interference” or “exposure”; C (comparison): comparison among children with C-BI and children without C-BI. O (outcome): different clinical results; S (design of examination): planned valuation was unlimited [10]. By using keywords in Table 1, we led a thorough exploration of applicable databases through May 2024. Appraisals were conducted on entire articles encompassed in a reference management program, comprising Author, titles, and abstracts. Also, two authors assess publications to detect appropriate tests [11]. 

**Table 1:** Database search strategy for inclusion of examinations

Database	Search strategy
Google Scholar	#1 “children"OR “asthma-connected emergency department visits” #2 “duration of asthma symptoms” OR"community‑based intervention” OR"“hospitalizations” OR “nighttime asthma symptoms” #3 #1 AND #2
Embase	#1 ‘children'/exp OR ‘asthma-connected emergency department visits’/exp OR ‘hospitalizations’ #2 ‘duration of asthma symptoms’/exp OR'community‑based intervention’/exp OR ‘nighttime asthma symptoms’ #3 #1 AND #2
Cochrane library	#1 (children): ti, ab, kw (asthma-connected emergency department visits): ti, ab, kw (hospitalizations): ti, ab, kw (Word variations have been searched) #2 (duration of asthma symptoms): ti, ab, kw OR (community‑based intervention): ti, ab, kw OR(nighttime asthma symptoms): ti, ab, kw (Word variations have been searched) #3 #1 AND #2
Pubmed	#1 “children “[MeSH] OR “asthma-connected emergency department visits“[MeSH] OR “hospitalizations” [All Fields] #2 “duration of asthma symptoms“[MeSH Terms] OR"community‑based intervention“[MeSH] OR “nighttime asthma symptoms “[All Fields] #3 #1 AND #2
OVID	#1 “asthma“[All Fields] OR “asthma-connected emergency department visits” [All Fields] OR “hospitalizations” [All Fields] #2 “duration of asthma symptoms“[All fields] OR “children with community‑based intervention“[All Fields] or “nighttime asthma symptoms“[All Fields] #3 #1 AND #2

### Research risk of bias assessment

The possibility of bias in the research and the caliber of methods used in publications selected for further analysis were examined by the author. Each test’s methodology was objectively reviewed by the author. The author appraised the technique of elect studies to decide the potential for their bias. Technical quality was judged utilizing the “risk of bias instrument” from Cochrane Handbook for Systematic Reviews of Interventions, Version 5. 1. 0 [12]. Upon classification of each study based on the assessment criteria, it was assigned one of the following bias risks: Research was classified as having a low bias risk if all criteria were satisfied, and as having a medium bias risk if one or more quality criteria were unmet. Research was considered to have a substantial risk of bias if several quality requirements were met either completely or partially.

### Screening of studies

The investigation is given in a standard style, along with all of its parts. Some of the things that were used to narrow down the data were the first author’s last name, the study’s date, the country where it took place, the gender of the women who took part, the total number of subjects, their clinical and treatment characteristics, demographic information, and the qualitative and quantitative evaluation methods that were used [13]. Two authors looked at the possibility of bias in studies and the quality of the methods used in the papers that were chosen for further study. There were two writers who looked at the methods used for each assessment without bias [14]. 

### Statistical analysis

This meta-analysis evaluated odds ratios (ORs) and mean differences (MDs) with a 95% confidence interval (CI) using dichotomous random or fixed-effect models. The calculated I2 index ranges from 0 to 100 and is represented as a percentage. Elevated I2 values indicate heightened heterogeneity, whereas diminished I2 values signify reduced heterogeneity. If I2 was 50% or greater, a random effect was picked; otherwise, a fixed effect was utilized [15]. The implications of the initial investigation were categorized as part of the subcategory analysis. Bias was assessed by Egger’s tests employed for quantitative analysis, and it was deemed present if *p* > 0.05 [16, 17]. *p*-values were computed using a two-tailed method. Graphs and statistical analyses were generated using Review Manager 5.4 [18]. 

## Results

After examining 2443 pertinent publications, 13 studies that were published between 2006 and 2024 satisfied requirements and were encompassed in this study [19–31]. 

Table 2 condenses the discoveries of these studies. 8824 persons were studied.

**Table 2:** Characteristics of studies

Study	Country	Total	Children with C-BI	Children without C-BI	Asthma severity	Age	Care provider
Portnoy, 2006 [19]	USA	186	93	93	Moderate or Severe	1 year	Psychologists, social workers
Thyne, 2006 [20]	USA	130	65	65	Not stated	3 years	Nurses
Fox, 2007 [21]	USA	560	280	280	Moderate or Severe	3 years	Nurses
Lob, 2011 [22]	USA	1522	761	761	Severe	6 years	Psychologists, social workers
Mansfield, 2011 [23]	USA	1443	720	723	Moderate or Severe	1 year	Nurses, asthma educators, asthma care facilitators
Findley, 2011 [24]	USA	1448	724	724	Not stated	1 year	Nurses
Woods, 2012 [25]	USA	566	283	283	Moderate or Severe	2 years	Nurses, asthma educators, asthma care facilitators
Lara, 2013 [26]	USA	262	117	145	Moderate or Severe	5 years	Psychologists, social workers
Turyk, 2013 [27]	USA	436	218	218	Not stated	1 year	Nurses
Janevic, 2016 [28]	USA	1610	805	805	Moderate or Severe	4 years	Nurses
Rapp, 2018 [29]	USA	374	187	187	Severe	3 years	Nurses, asthma educators, asthma care facilitators
Elliott, 2022 [30]	USA	100	50	50	Moderate or Severe	1 year	Nurses
Basnet, 2024 [31]	USA	187	123	64	Not stated	1 year	Psychologists, social workers
	**Total**	**8824**	**4426**	**4398**			

**Fig. 2:**
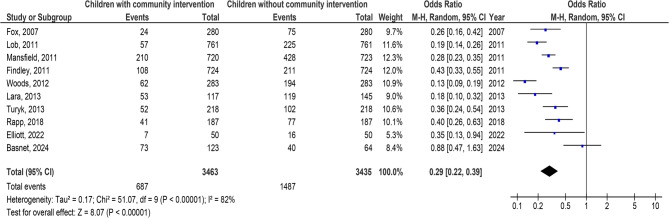
Effect’s forest plot of children with community intervention on asthma-connected emergency department visits compared to children without community intervention in children with asthma

**Fig. 3:**
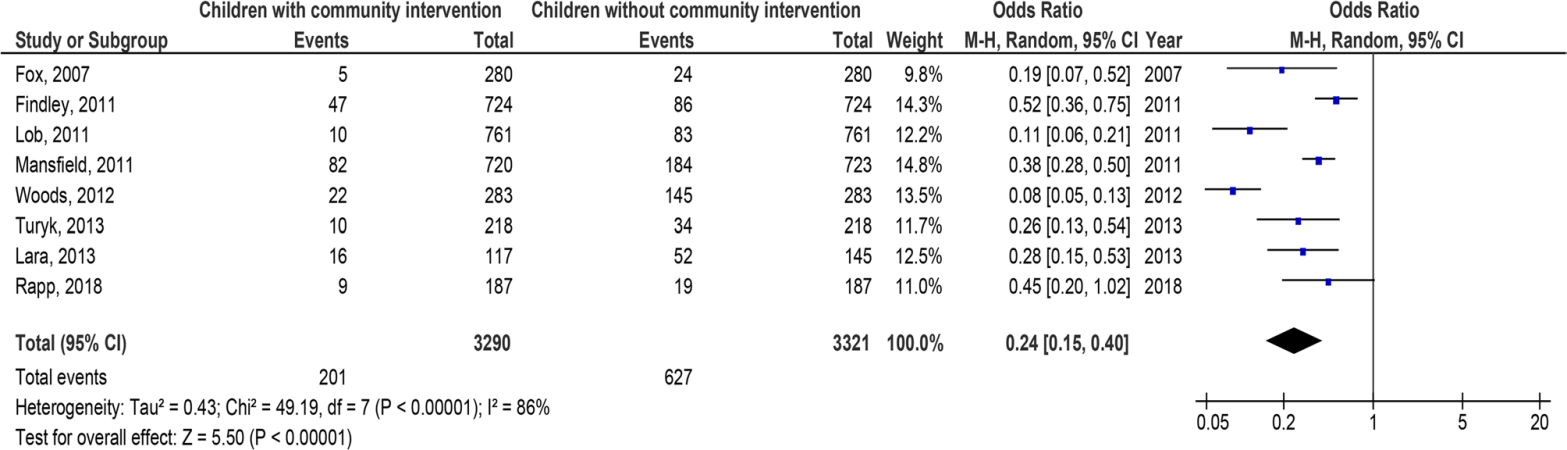
Effect’s forest plot of children with community intervention on hospitalizations compared to children without community intervention in children with asthma

**Fig. 4:**
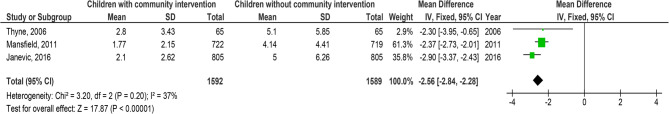
Effect’s forest plot of children with community intervention on duration of asthma symptoms compared to children without community intervention in children with asthma

**Fig. 5:**
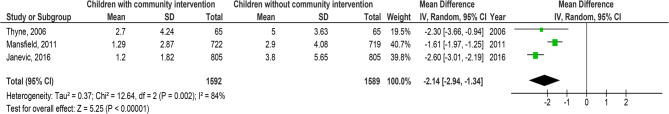
Effect’s forest plot of children with community intervention on nighttime asthma symptoms compared to children without community intervention in children with asthma

**Fig. 6:**
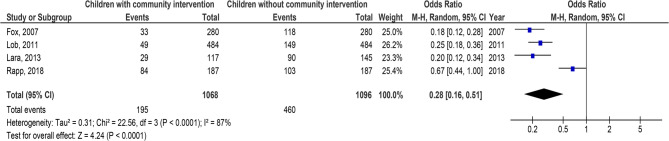
Effect’s forest plot of children with community intervention on uses of bronchodilators compared to children without community intervention in children with asthma

**Fig. 7:**
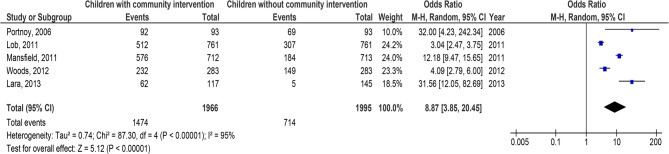
Effect’s forest plot of children with community intervention on asthma action plan compared to children without community intervention in children with asthma

**Fig. 8:**
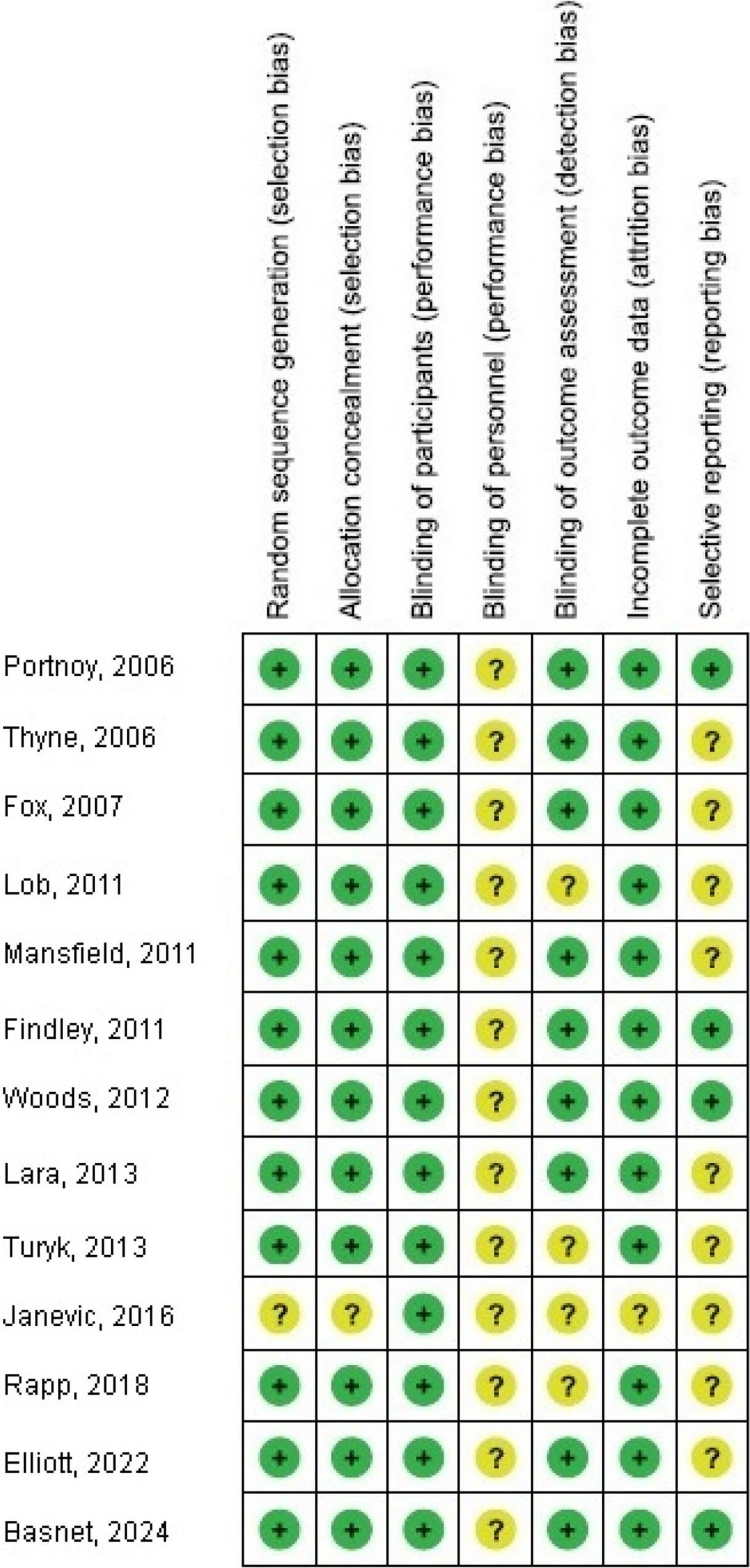
Risk-of-bias plot

Children with C-BI had significantly lower asthma-connected emergency department visits (OR, 0.29; 95% CI, 0.22–0.39, *p* < 0.001) with high heterogeneity (I^2^ = 82%), hospitalizations (OR, 0.24; 95% CI, 0.15–0.40, *p* < 0.001) with high heterogeneity (I^2^ = 86%), duration of asthma symptoms (MD, −2.56; 95% CI, −2.84- −2.28, *p* < 0.001) with low heterogeneity (I^2^ = 37%), nighttime asthma symptoms (MD, −2.14; 95% CI, −2.94- −1.34, *p* < 0.001) with high heterogeneity (I^2^ = 84%), and use of bronchodilators (OR, 0.28; 95% CI, 0.16–0.51, *p* < 0.001) with high heterogeneity (I^2^ = 87%), and higher use of asthma action plan (OR, 8.87; 95% CI, 3.85–20.45, *p* < 0.001) with high heterogeneity (I^2^ = 95%) compared to children without C-BI in asthma, as shown in Figs. 2, 3, 4, 5, 6 and 7. There was no evidence of investigation bias (*p* = 0.86) in the quantitative Egger regression test or the visual reading of the effect’s forest plot. It was shown that most relevant tests were not very good in practice and were biased in the reports they gave as shown in Fig. 8.

## Discussion

For the current meta-analysis, 13 studies that were published between 2006 and 2024 were included; of these, 8824 people were studied [19–31]. 

Children with C-BI had significantly lower asthma-connected emergency department visits, hospitalizations, asthma symptoms, nighttime asthma symptoms, use of bronchodilator, and use of asthma action plan compared to children without C-BI in asthma. However comparisons comprised a small number of studies, and thoughtfulness ought to be prearranged to their values.

The goal of asthma management should be to achieve the best possible control over the condition while lowering the risk of serious consequences. Effective asthma management, like that of many other chronic illnesses, necessitates participation of numerous stakeholders, including the patient’s family, school, community, and policy organizations or agencies, to support the patient’s position as health manager [32, 33]. The chronic Care Model was developed by Wagner and colleagues to address shortcomings in the management of chronic diseases, including poor adherence to practice guidelines, an absence of care matching and planned care, an absence of active follow-up, and insufficient patient education [33]. The six elements of the Chronic Care Model are self-management support, community resources, decision support, delivery system design, organizational support, and clinical information systems. The most important of the six Chronic Care Model components is self-management, which entails emphasizing patients’ pivotal role in controlling their condition and offering patients and the families assistance they need to develop the knowledge and self-assurance necessary to oversee their medical treatment [32]. Our meta-analysis clearly shows that this is very important for asthma care, with one of the key core interventions being education on fundamental asthma knowledge and self-management skills. To achieve the best possible asthma control, an intervention approach that solely focuses on asthma education might not be sufficient. Researchers recently found some improvement in caregivers’ knowledge and skills about asthma, but no discernible improvement in unscheduled medical visits or quality of life for Aboriginal children with asthma who participated in a community outreach program focused on asthma education [34]. According to a review by Wu & Takaro [35], studies that used asthma education alone did not show these kinds of improvements in asthmatic children’s acute service utilization or clinical symptoms. However, a combination of interventions, including environmental measures for trigger decrease and self-management skill education, did. Prior research on educational programs for asthma self-management has also been the subject of several meta-analyses, however, findings were mixed [5, 36], indicating that asthma education might not be enough on its own to effectively manage asthma, particularly in young patients. Among the studies in this analysis, home visits and assessments of environmental triggers are two more often employed therapies. Asthma exacerbations are associated with indoor dwelling conditions and exposure to allergens, including bugs, ambient tobacco smoke, and poor ventilation [37]. During home visits, possible environmental factors for asthma exacerbations can be identified and addressed. The effectiveness of treatments aimed at reducing asthma triggers in the home has been assessed in numerous trials. While studies regularly documented improvements in asthma symptoms and fewer emergency department visits. The degree of asthma control and hospital admissions for asthma remained unclear [38]. Additionally, to remove potential triggers from the home environment, families receiving home-based therapies were frequently given environmental remediation goods e.g. vacuum cleaners, bed coverings, and insect abatements. According to several suggestions, the provision of environmental remediation goods might not yield a greater advantage in terms of asthma outcomes [39]. This agrees with our findings in part. To help patients or families navigate health care or social systems, care coordination is a critical component. It makes communication easier between families and medical professionals as well as connections to social services for family needs and issues. Care coordination, along with self-management education and home environmental valuation, is one of the main interventions in all of the asthma programs that we have studied here. A noteworthy decrease in emergency department visits and hospital admissions was consistently found previously in studies that focused specifically on care coordination; this suggests the significance of care coordination in enhancing health outcomes for children with asthma. Future studies should examine whether care coordination on its own is sufficient to attain successful asthma control, as this has not yet been established [23, 24, 28]. The majority of reviewed studies aimed at promoting asthma awareness and support, as well as advocating for more asthma-friendly environments or policy changes, expanded their interventions beyond core strategies to include schools, primary care practices, public organizations, and the broader community. There were significant differences in components of interventions of remaining studies, even though nine studies exhibited analogous program designs encompassing all seven intervention aspects, specifically the three main components along with the engagement of schools, primary care practitioners, community campaigns, and policymakers/organizations, among others [24, 28]. As a result, it is impossible to pinpoint a precise mix of intervention components that produced the improvement seen in summary effects. It was also noteworthy that usage of telehealth care, which has received a lot of attention in the wake of the COVID-19 pandemic, was not included in any of the studies. Telehealth, as a term, often describes health services that are provided via electronic communication channels e.g. Internet, video, or phone. It has been demonstrated to be cost-efficient in giving patients with chronic illnesses health advice and education, and it is effective in overcoming geographical obstacles [40]. However, there was no discernible increase in patients’ quality of life or emergency department visits for those with asthma, according to a latest meta-analysis of 21 randomized controlled studies using telehealth therapy including text messaging, phone calls, video conferences, etc [41]. Further studies are required to validate effect of telemedicine on children’s asthma management. There are a few things to be aware of with this review. Randomized controlled studies are hard to come by for multimodal community-based therapies, especially when it comes to childhood asthma. Low number of included studies and varied study designs, contexts, and intervention components likely contributed to the high amount of heterogeneity that was found. To integrate between-study variability, random effect models were employed to determine the summary effect size. All studies included were carried out in the USA, and participants were primarily from low-income households and ethnic minorities. Findings of this analysis might not apply to other nations because of how unique USA healthcare system is.

Moreover, the bias assessment showed very low to intermediate methodological quality, which means we are incapable to make definitive judgments concerning the effect of C-BIs for childhood asthma. The evaluations in the certainty assessment were significantly affected by the domains of indirectness of evidence and risk of bias. The absence of information about allocation concealment all decreased the quality of evidence by one degree. Since we discovered a significant amount of variation in the outcomes for which we were incapable to find a reasonable explanation, the generalization result’s certainty assessment was also decreased by one notch for inconsistency. These findings support the need for more study and will possibly significantly affect our capacity to forecast the treatment’s efficacy. As an outcome, more high-quality studies and data about the effect of C-BIs for childhood asthma are required to enhance future research.

## Conclusions

Children with C-BI had significantly lower asthma-connected emergency department visits, hospitalizations asthma symptoms, nighttime asthma symptoms, use of bronchodilator, and use of asthma action plan compared to children without C-BI in asthma. However comparisons comprised a small number of studies, and thoughtfulness ought to be prearranged according to their values.

## Supplementary Information

Below is the link to the electronic supplementary material.

Supplementary Material 1

## Data Availability

All data generated or analyzed during this study are included in this published article.
